# Application of dermatoscopy, reflectance confocal microscopy, and high‐frequency ultrasound for diagnosing neonatal lupus erythematosus: A case report

**DOI:** 10.1111/srt.13291

**Published:** 2023-02-17

**Authors:** Yunjing Pu, Li Zhang

**Affiliations:** ^1^ Department of Dermatology, Kunming Children's Hospital Yunnan Key Laboratory of Children's Major Disease Research Kunming China

Dear Editor,

A 2‐month‐old female infant was admitted to the hospital for “annular erythema, scales, and trophy in facial area and body for 1 month.” The baby had no history of fever, external drug treatment, or oral drug therapy during the disease course. The infant's gestational age was 39 ^+^ ^3^ weeks, and her body weight was 3100 g at birth. She received breast feeding exclusively and had no history of intrauterine hypoxia or birth asphyxia. The prenatal examinations of her mother showed normal findings, who reported no history of gestational hypertension, diabetes, or syphilis. No relevant family history was reported. Dermatological examinations showed annular erythema with different sizes (range of dimeter, 0.5–1.5 cm) at facial area and body of the baby, of which the edges were slightly raised, and the color decreased when pressed. Fine yellow‐white scales were found in several skin lesions, and atrophy was detected at the center (Figure 1A,B). The infant's liver function examination revealed that the total bilirubin level was 21.5 (normal, 3.4–17.7) μmol/L, and the direct bilirubin level was 6.3 (normal, 0–3.4) μmol/L; in addition, the other indicators also showed no abnormality. Treponema pallidum antibody was negative (−), electrocardiograph showed nodal tachycardia (heart rate, 156 bpm). Echocardiography displayed patent foramen ovale (approximately 3.5 mm). Examination of antinuclear antibodies (ANA) in the baby revealed the following results: ANA (−), natural anti‐Sjogren's syndrome A 60kDa (SSA‐60) (++), anti‐52 kDa human Ro ribonucleoprotein antibody (Ro‐52)(−), anti‐Sjogren's syndrome B antiboy (SSB antibody) (−), and anti‐doubl‐strand deoxyribonucleic acid (ds‐DNA antibody) (−). The mother reported no clinical manifestations of connective tissue diseases, such as Sjogren's syndrome (SS) and systemic lupus erythematosus (SLE), and the results of ANA examination could be summarized as follows: ANA (+, titer 1:80), extractable nuclear antigens (ENA antibody) (+), Ro‐52 antibody (−), SSA‐60 antibody (+), SSB antibody (−), and ds‐DNA antibody (−). The patient's dermoscopy showed light red background, disappearing of skin surface texture, and whitish structureless areas, and dispersed irregular linear or reticular vascular structures (Figure 2). Reflectance confocal microscopy (RCM) revealed focal spongiotic edema in the spinous layer of skin lesion, and inflammatory cells with the high refraction migrated to epidermis. The dermal‐epidermal junction was full of inflammatory cells, which made the junction unclear. Besides, hemangiectasis and infiltration of various highly refractive inflammatory cells were found at the level of the superficial dermis (Figure 3). High‐frequency ultrasound (HFUS) examination showed that the epidermis was thickened, and a hypoechoic zone appeared at the dermal‐epidermal junction and superficial dermis, and the boundary was unclear (Figure 4). Combined with clinical and laboratory tests, the infant was diagnosed with neonatal lupus erythematosus (NLE). Due to the laboratory tests and her physical condition, we only required the baby strict sun protection without any special treatment. Five months later, the lesion was disappeared (Figure 1C), and her antinuclear antibody examination was negative.

**FIGURE 1:**
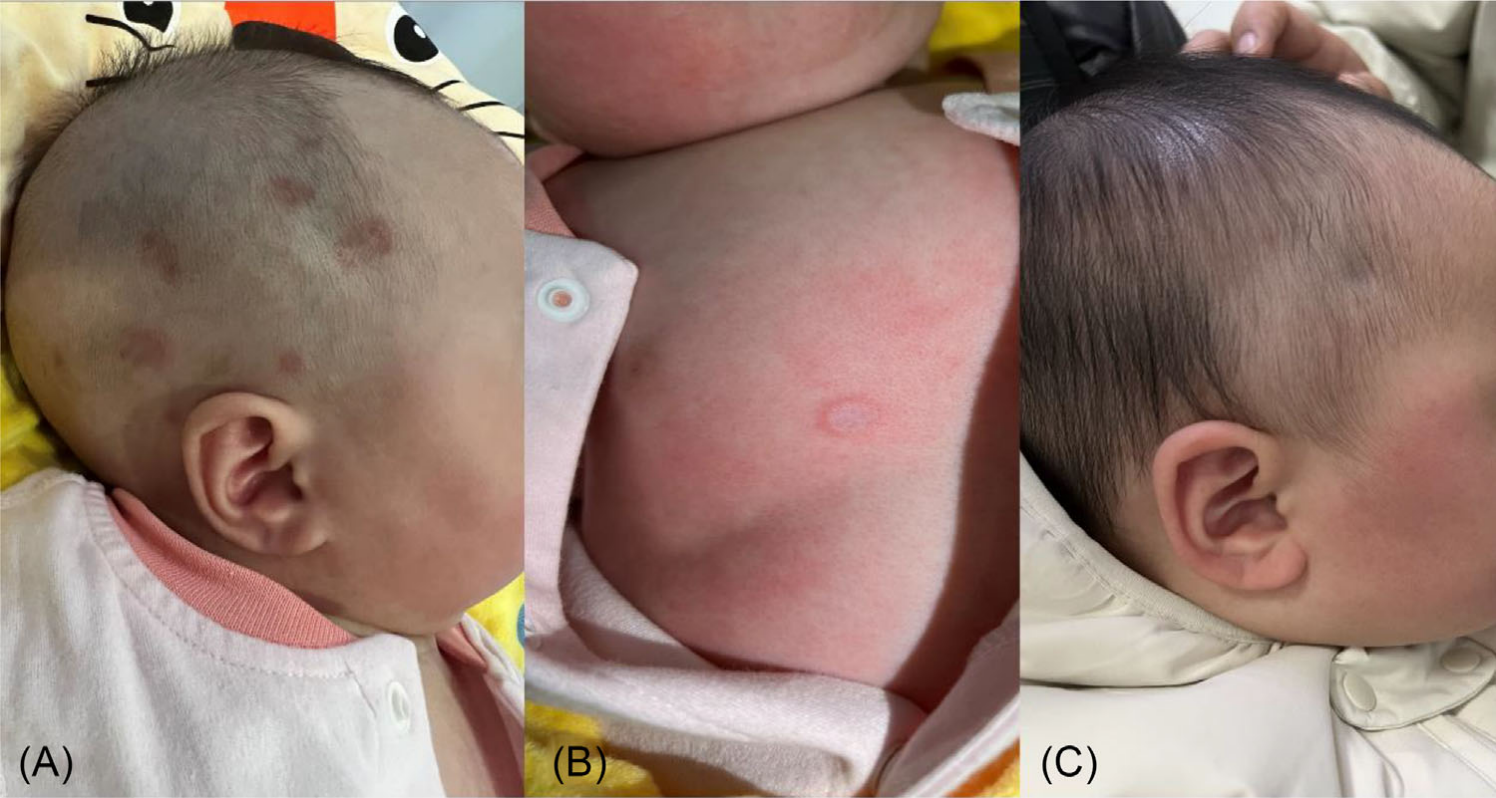
Clinical manifestation of the neonatal lupus erythematosus (NLE). A and B Annular erythema with various sizes at the facial area and body of the baby at her first visit, with the edges that were slightly raised. Fine yellow‐white scales were found in several skin lesions, of which the center was found with depression and atrophy. C All the lesions at her facial area faded away after 5 months later.

**FIGURE 2:**
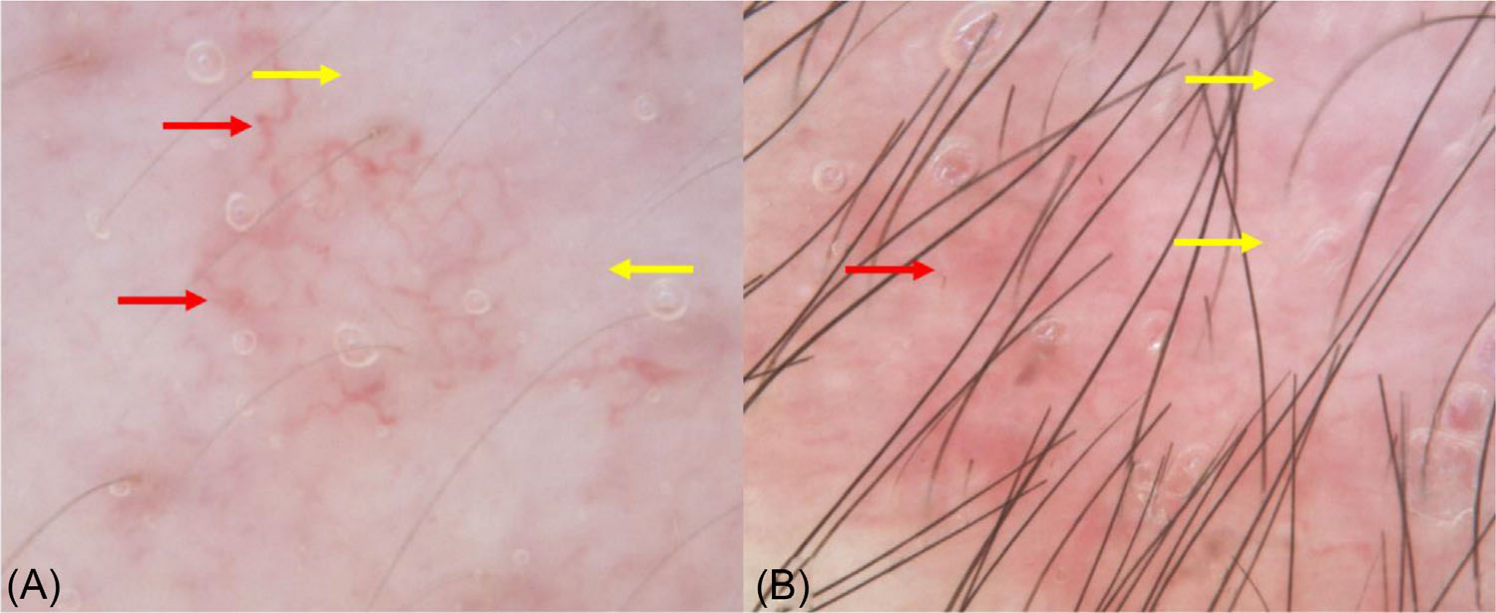
Dermatoscopy of the neonatal lupus erythematosus (NLE) showed hemangiectasis (red arrows), as well as disappearing of texture on skin surface, and whitish structureless areas (yellow arrows) (A and B: 50×).

**FIGURE 3:**
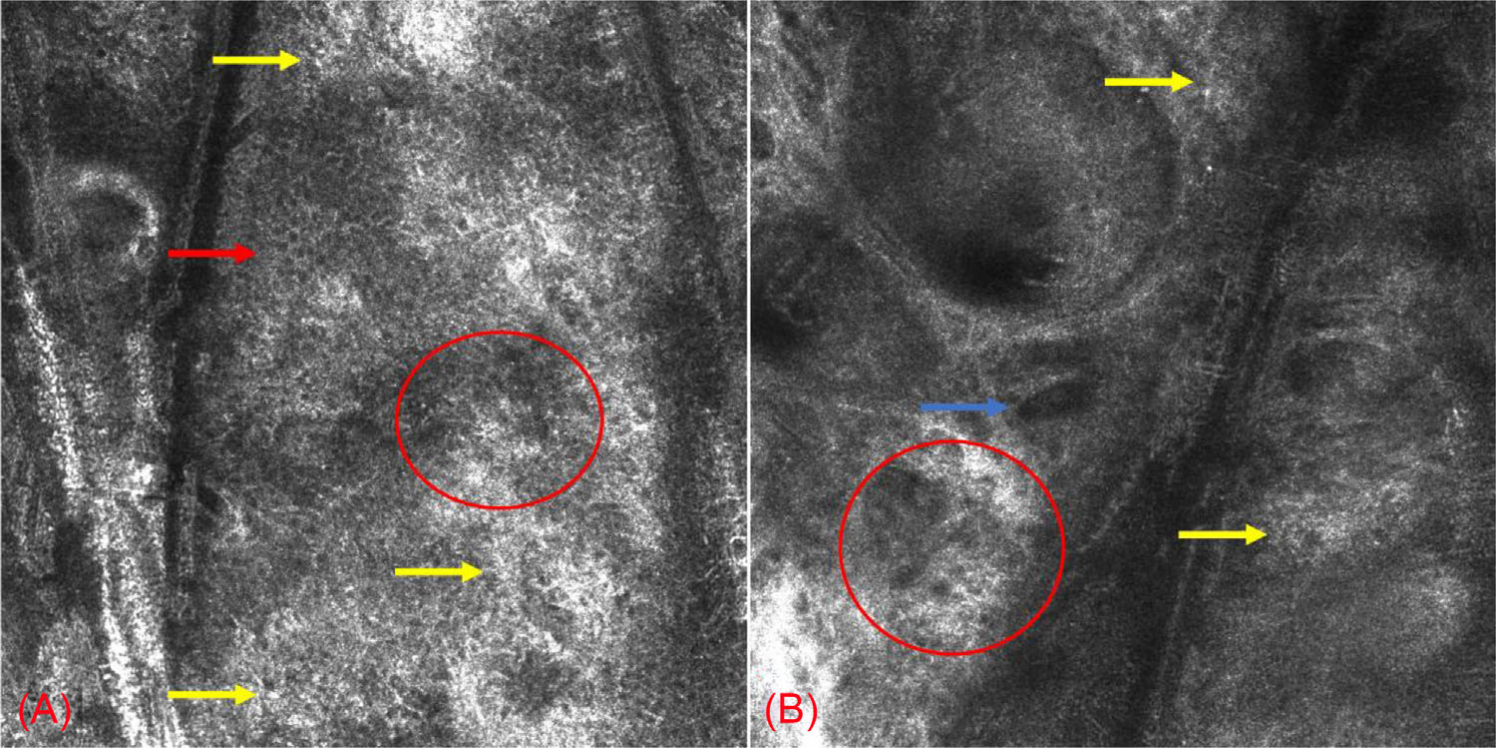
Reflectance confocal microscopy showed slight edema in the spinous layer of skin (red arrow), highly refractive inflammatory cells (yellow arrows) and unclear dermal‐epidermal junction (red circles), and hemangiectasis (blue arrow).

**FIGURE 4:**
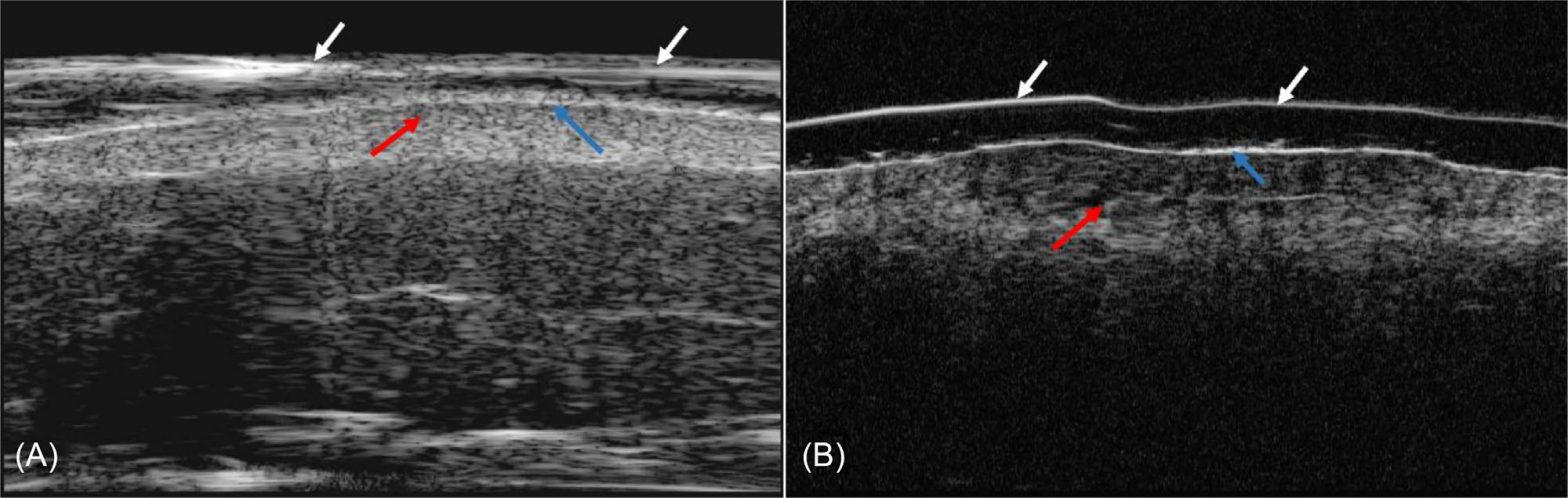
High‐frequency ultrasound examination revealed a hypoechoic zone at the dermal‐epidermal junction and superficial dermis (red arrows),thickened epidermis (blue arrows). Foil membrane (white arrows). (A: 20 Hz, B: 50 Hz).

It is noteworthy that NLE is a rare autoimmune disease with the incidence of approximately 1/20 000,^1^ which was firstly reported by McCuistion and Schoch in 1954. At present, it is generally considered that maternal lupus‐associated autoantibodies are passively transferred to fetus through placenta.^2^ Although 30%–50% of mothers have no symptoms before delivery, SSA or SSB antibody is detected in maternal serum.^3^ In some cases, the fetal antibodies are not fully consistent with maternal antibodies, and several studies reported the delivery of babies with NLE from mothers with negative antibodies. These findings indicate that maternal antibody transmission is not the only cause of NLE.^3^ The baby reported here was found with annular erythema of different sizes at facial area, scalp, and body at 1 month after birth. Although no clinical symptom was found in the mother, serum SSA‐60 antibody in both baby and mother was positive, which was in agreement with the American College of Rheumatology (ACR) diagnostic criteria for NLE (2014 edition).^4^ SSA antibody includes two subtypes of SSA‐60 and SSA‐52/TRIM21 antibodies, while SSA‐60 antibody is closely associated with SLE and SS. In addition, SSA‐60‐positive antibody could be the only indicator in some cases of SLE, which has especially a high specificity in patients with cutaneous lupus erythematosus.^5^

The pathological manifestations of NLE are similar to subacute lupus erythematosus, mainly including epidermal atrophy, liquefaction of basal cells, and dermal and perivascular infiltration of lymphocytes.^6^ Due to the possible risks of anesthesia and surgery, this baby's family refused pathological examination. Therefore, dermatoscopy, RCM, and HFUS examinations were used for the assessment of skin lesions in the baby. Dermatoscopy showed disappearing of texture on skin surface and whitish structureless areas, as well as focal hemangiectasis. HFUS examination revealed a hypoechoic zone at the dermal‐epidermal junction and superficial dermis. RCM showed unclear dermal‐epidermal junction, hemangiectasis, and infiltration of various highly refractive inflammatory cells at the level of epidermis, dermal‐epidermal junction and superficial dermis. The manifestations of the three measurements were highly in agreement with the pathological manifestations of NLE. To date, no study has concentrated on skin imaging in NLE patients. As pathological examinations could not be performed for newborns, noninvasive examinations by dermatoscopy, RCM, and HFUS have a high consistency with histopathological examinations and play important roles in the early diagnosis and differential diagnosis of NLE.

## CONFLICT OF INTEREST

The authors declare that there is no conflict of interest.

## FUNDING INFORMATION

Yunnan Key Laboratory of Children's Major Disease Research; Yunnan Province Clinical Research Center for Children's Health and Disease

## Data Availability

Data are available upon request from the authors.
